# Improvement in the Thermostability of a Recombinant β-Glucosidase Immobilized in Zeolite under Different Conditions

**DOI:** 10.3390/molecules27134105

**Published:** 2022-06-26

**Authors:** Luis Gerardo Ramírez-Ramírez, David Enrique Zazueta-Álvarez, Héctor Alonso Fileto-Pérez, Damián Reyes-Jáquez, Cynthia Manuela Núñez-Núñez, Juan de Dios Galindo-De la Rosa, Javier López-Miranda, Perla Guadalupe Vázquez-Ortega

**Affiliations:** 1 Departamento de Ingenierías Química y Bioquímica, Tecnológico Nacional de México (TecNM)—Instituto Tecnológico de Durango (ITD), Durango 34080, Mexico; 17040401@itdurango.edu.mx (L.G.R.-R.); hfileto@itdurango.edu.mx (H.A.F.-P.); damian.reyes@itdurango.edu.mx (D.R.-J.); jlopez@itdurango.edu.mx (J.L.-M.); 2 Departamento de Ingeniería en Tecnología Ambiental, Universidad Politécnica de Durango, Durango 34300, Mexico; david.zazueta@unipolidgo.edu.mx (D.E.Z.-Á.); cynthia.nunez@unipolidgo.edu.mx (C.M.N.-N.); 3 División de Investigación y Posgrado, Facultad de Ingeniería, Universidad Autónoma de Querétaro, Centro Universitario, Querétaro 76010, Mexico; juandedios_galindo@hotmail.com

**Keywords:** enzyme immobilization, β-glucosidases, zeolite, enzyme stability

## Abstract

β-Glucosidase is part of the cellulases and is responsible for degrading cellobiose into glucose, a compound that can be used to produce biofuels. However, the use of the free enzyme makes the process more expensive. Enzyme immobilization improves catalytic characteristics and supports, such as zeolites, which have physical-chemical characteristics and ion exchange capacity that have a promising application in the biotechnological industry. This research aimed to immobilize by adsorption a recombinant β-glucosidase from *Trichoderma reesei*, obtained in *Escherichia coli* BL21 (DE3), in a commercial zeolite. A Box Behnken statistical design was applied to find the optimal immobilization parameters, the stability against pH and temperature was determined, and the immobilized enzyme was characterized by SEM. The highest enzymatic activity was determined with 100 mg of zeolite at 35 °C and 175 min. Compared to the free enzyme, the immobilized recombinant β-glucosidase presented greater activity from pH 2 to 4 and greater thermostability. The kinetic parameters were calculated, and a lower K*_M_* value was obtained for the immobilized enzyme compared to the free enzyme. The obtained immobilization parameters by a simple adsorption method and the significant operational stability indicate promising applications in different fields.

## 1. Introduction

Second-generation (2G) ethanol is a promising substitute for fossil fuels, as well as an additive for gasoline that can improve oxygenation and performance, thus reducing the dependence on non-renewable energy resources [1]. Lignocellulosic biomass is the most abundant source of glucose; however, to obtain it, it is necessary to degrade cellulose [2]. Cellulases are enzymes that participate in the hydrolysis of cellulose by breaking the β-1,4-glucosidic bond. The cellulolytic complex is formed by three enzymes: endoglucanase, exoglucanase, and β-glucosidase [3]. β-glucosidase (E.C. 3.2.1.21) is an enzyme that acts on the β-1,4 bonds that join two glucose or molecules with glucose substitutions (such as disaccharide cellobiose). It is an exo cellulase with specificity for several substrates of β-D-glucosides that catalyzes the hydrolysis from waste terminals, which, without β-D-glucosides reducers, produces glucose [4], a compound that can be used in the biofuels industry and in the production of fine chemicals and medicines [5]. Thus, they are also used to enhance the release of some glycosylated substances (such as aromas in wine and other products) [6] or the production of some glycosyl bonds [7]. β-glucosidase can be produced by different organisms, including bacteria and fungi [8]. To improve the yield of cellulase production and reduce the cost of hydrolysis, several techniques have been implemented, such as the production of recombinant enzymes expressing genes in more efficient organisms, such as *E. coli* [9]. One of the most used hosts to produce recombinant proteins is *E. coli* BL21 (DE3), which is induced by isopropyl β-D-1-thiogalactopyranoside (IPTG) [10]. However, the use of β-glucosidase in soluble form has some disadvantages, such as its low stability and the difficulty of recovering the enzyme to use it in consecutive cycles, which is one of the main challenges that must be faced in biocatalysts to decrease the high cost of enzymes [11].

One strategy to overcome these limitations is the immobilization of the enzyme on physical support; this process allows to take advantage of heterogeneous catalysis, such as the possibility of reusing the biocatalyst and increasing the stability of the enzymes against changes in temperature and pH, according to the applied immobilization protocol [12,13]. During immobilization, some parameters must be considered, such as enzyme selection, type of support, and immobilization conditions such as temperature, pH, ionic strength, and time.

Immobilization methods are crosslinking, adsorption, encapsulation, ion exchange, affinity, and covalent bonding [14]. Immobilization by adsorption (anion exchange) to porous material supports has proven to be a promising strategy for obtaining active and stable biocatalysts [15,16]. The simple adsorption immobilization process is gentle and does not require the use of toxic chemical reagents, so it has more benefits compared to other immobilization methods to maintain stability against environmental changes and catalytic activities of enzymes [17]. The supports to immobilize by adsorption are silica gel, magnetic nanomaterials, and zeolites.

In recent years, zeolites have been considered ideal support for enzyme immobilization because of their high retention of enzymes and have a large surface area ideal for reduction and oxidation reactions, which allows the adsorbed enzymes to have adequate immobilization [18,19]. Zeolites are microporous aluminosilicate minerals with SiO_4_ and AlO_4_, which are connected by shared oxygen atoms [20]. However, one of the unavoidable disadvantages is that the interaction between the enzyme and the zeolite is affected by the ionic strength and the solvent used for immobilization, which leads to the desorption of the enzyme, and its use is limited to a few reaction cycles [18,21]. Therefore, different strategies have been proposed to improve the affinity of the enzyme with support, for example, through adsorption and selectivity affinity tags that fuse at the carboxyl or amino terminus of the protein [22].

The β-glucosidase used for this research is specifically designed with affinity tags; a 6-lysine tag at the *C* terminal does not reduce enzymatic activity during the immobilization process, and a 6-histidine tag at the *N* terminal purifies the enzyme by nickel-agarose column affinity chromatography. These two tags contribute to improving the affinity between the enzyme and the support and, consequently, increase the stability of the immobilized enzyme. In this research, the main objective was to immobilize by adsorption recombinant β-glucosidase obtained from the *E. coli* BL21 (DE3) strain and to study the effect of the amount of support, the temperature, and the immobilization time in a commercial zeolite and to evaluate its stability under different pH and temperature conditions.

## 2. Materials and Methods

Reagents, solvents, and culture media of analytical degree were purchased from Sigma-Aldrich (St. Louis, MO, USA), and zeolite was purchased from Merck (Darmstadt, Germany). To produce the recombinant β-glucosidase *E. coli* BL21 (DE3) (GIBCO, Darmstadt, Germany) was used and transformed with the plasmid pET-28a-β-glusy [23].

### 2.1. Production and Recovery of the Recombinant β-glucosidase

β-glucosidase was obtained from a fresh culture of the recombinant *E. coli*-pET-28a-β-glusy as described by Vázquez-Ortega et al. [23] as follows: After centrifugation in a refrigerated centrifuge (HERMLE z 326 k), cells were placed on ice and suspended in 0.1 M acetate buffer (pH 5). With an ultrasonic processor (Hielscher UP200Ht), cells were disrupted with 10 pulses of 10 s separated by a 30 s pause at a 40% amplitude. Enzymatic activity and protein concentration were measured in the supernatant after centrifugation in a refrigerated centrifuge (HERMLE z 326 k) at 3175× *g* at 4 °C for 15 min. The soluble fraction of proteins was verified by SDS-PAGE analysis using a Miniprotean III System (BioRad, Hercules, CA, USA). Proteins were stained with Coomassie blue R-250 (BioRad) (Hercules, CA, USA). The clarified lysate was purified by affinity chromatography on a His 6 Ni Superflow Resin column (Clontech, Mountain View, CA, USA) of 10 mL, following the supplier’s specifications.

### 2.2. Enzymatic Activity and Protein Concentration

The activity was determined for both free and immobilized enzymes by a spectrophotometric method: 5 mg of zeolite/β-glucosidase or 100 µL of the diluted pure enzyme were incubated in 0.1 M acetate buffer (pH 5; 2 mL final volume), and 800 µL of 5 mM *p*-nitrophenyl-β-D-glucopyranoside (*p*-NPG) for 10 min at 50 °C. The reaction was stopped with 3 mL of sodium hydroxide (NaOH), and the immobilized enzyme was centrifugated at 3175× *g* at 4 °C for 10 min, after which aliquots of the supernatants were taken, and their absorbance at 385 nm was measured in a spectrophotometer (DR 6000 of HACH, Loveland, CO, USA). The concentration of the purified protein was determined by the Bradford method using bovine serum albumin as a standard [24].

### 2.3. Immobilization of Recombinant β-glucosidase on Commercial Zeolite

The immobilization by adsorption was carried out as follows: 25 mg of commercial zeolite were mixed with 3 mL of β-glucosidase enzymatic solution (10 mg/mL) in 0.1 M sodium acetate at pH 5 and was incubated at 25 °C for 240 min, with constant stirring at 6× *g*. A parallel blank experiment was carried out using a free enzyme to compensate for enzyme deactivation under immobilized conditions. Periodically, samples of supernatant were taken after centrifugation, and the suspension samples were collected under agitation, and the enzymatic activity was evaluated. A Box Benhken response surface experimental design was applied to evaluate three immobilization parameters: the amount of support (mg), temperature (°C), and time (min) (Table 1). After immobilization, the immobilized β-glucosidase was washed and resuspended in the same buffer. Protein concentration was evaluated in the initial, final, and washing solutions. Relative activity during the immobilization process and the immobilization yield were calculated from Equations (1) and (2) [25].

Relative activity (%) = 100 (Detected activity)/(Starting activity)(1)

Immobilization yield (%) = 100 (Initial protein concentration − Final concentration plus that of wash solutions)/(Initial protein concentration)(2)

### 2.4. Characterization of Free and Immobilized β-glucosidase

#### 2.4.1. Effect of pH and Temperature on Enzyme Stability

To determine the stability against exposure to different pH conditions on the activity of free and immobilized recombinant β-glucosidase, aliquots of 100 µL were incubated in 1 mL of different buffers at different pH values (from 2 to 10) at 4 °C for 12 h. After incubation, immobilized β-glucosidase was removed by filtration; subsequently, residual activity (%) was determined according to the described technique, and it was expressed as relative activity (%), concerning maximum activity, which was considered as 100%. The effect of temperature on the stability of free and immobilized recombinant β-glucosidase was determined by using aliquots of the enzyme, which were heated at different temperatures (20–80 °C) for 240 min. The residual activity was determined at 0, 30, 60, 90, 120, 180, and 240 min according to the described technique, and the initial activity was considered as 100%, and the following values of residual activity were expressed with this value as a reference. All the experiments were carried out in duplicate.

#### 2.4.2. The Kinetic Parameters of the Enzyme

The Michaelis–Menten model and Lineweaver–Burk method were applied to determine the kinetic parameters of the enzyme. The activity was measured using 1.1 mL sodium acetate buffer (0.1 M, pH 5), 0.1 mL of free or immobilized enzyme solution, and different concentrations of *p*-NPG (1–10 mM) as substrate. Optimum reaction conditions (pH 5, 50 °C) were used, and the K*_M_*, V*_max_*, and K_cat_ of the free and immobilized β-glucosidase were obtained. All the experiments were carried out in triplicate.

#### 2.4.3. Reutilization of Immobilized β-glucosidase

The reusability of immobilized β-glucosidase was evaluated by submitting the supported biocatalyst to consecutive reaction cycles, measuring enzyme activity as previously specified. After each cycle, the immobilized β-glucosidase was separated from the reaction mixture by centrifuging at 3913× *g* for 2 min. The absorbance of the supernatant was noted with a spectrophotometer at 385 nm. Then, the immobilized enzyme was washed and reused in a fresh reaction buffer under the same reaction conditions. The initial enzyme activity in the first cycle was considered 100%. All the experiments were carried out in triplicate, and the results are expressed as the means SD.

### 2.5. Microstructural Analysis

The morphology and size of immobilized β-glucosidase and the zeolite were determined by electron scanning micrographs using a Hitachi scanning electron microscope (SEM) model SU3500 located at the Technological University of San Juan del Rio. Before their observation, the immobilized β-glucosidase and the zeolite were stored in a vacuum desiccator. Operating with a voltage of 5 and 10 kV. The samples were coated with layers of gold using coating spray equipment to create better conduction before SEM imaging. Secondary electron mode was used to capture the photos at various magnifications.

## 3. Results and Discussion

### 3.1. Production of Recombinant β-glucosidase

Recombinant β-glucosidase was successfully expressed in soluble form in *E. coli* BL21 (DE3) after induction with isopropyl β-D-1-thiogalactopyranoside (IPTG). The β-glucosidase crude extract was obtained as the supernatant after the centrifugation of a sonicated fresh culture of the recombinant *E. coli*-pET-28a-βglusy, as explained in Section 2.1. Later, the enzymatic crude extract was characterized concerning its protein concentration, protein electrophoresis analysis on a 12% SDS-PAGE gel, and enzymatic activity. The protein concentration in the crude extract was 37.68 ± 1.84 mg/mL, and the enzymatic activity was 8.70 ± 1.32 U/mL. An activity of 23.72 ± 3.40 U/mg was reached for pure recombinant β-glucosidase.

### 3.2. Immobilization by Adsorption of Recombinant β-glucosidase on a Commercial Zeolite

#### 3.2.1. Immobilization Process

**Figure 1:**
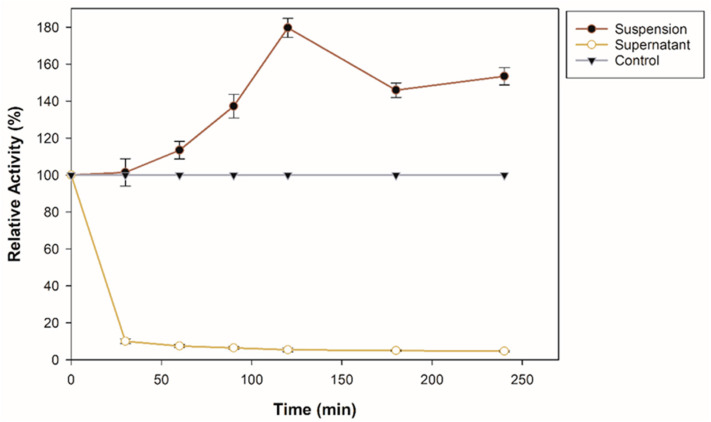
Immobilization course of recombinant β-glucosidase on zeolite at pH 8, 25 °C, and shaking.

An immobilization assay was performed to investigate the adsorption capacity of commercial zeolite for β-glucosidase. The enzyme solution was mixed with 25 mg of support. Figure 1 shows the immobilization course at pH 5 for 240 min. As observed, the suspension, that is, the β-glucosidase bound to the support, increases its enzymatic activity, more than 70% of its initial activity. An immobilization yield of 86.97% was obtained after 240 min. It is important to emphasize that the β-glucosidase used in this research contains a lysine tag and, according to previous reports, an enzyme that contains lysine residues on its surface and is bound to a negatively charged surface such as zeolite generates strong electrostatic interactions [26]. In addition, the reason why β-glucosidase could increase its activity is also that the interactions may be through the alkaline amino acid tag (6-histidine) at the *N*-terminal [27]. Specific surface characteristics, surface area, and SiO_2_ content also influenced the adsorption affinity and enzyme activity after immobilization [19]. Techniques for the immobilization of β-glucosidase through interactions such as covalent bonds and encapsulation have been previously reported; for example, it has been immobilized on magnetic nanoparticles with a binding efficiency of 96.5% [28], on amino-based silica with an immobilization efficiency 5.86 times that of the free enzyme [14], on a new magnetic nanomaterial of MnO_2_ exhibiting greater thermal stability than the free one [29], in SiO_2_ nanoparticles crosslinked with glutaraldehyde with an immobilization yield of 83.34% [30], on CNBr-activated Sepharose [31]. However, in this study, it is important to highlight that the increase in enzymatic activity during the immobilization process is related to the improvement of the adsorption affinity in the zeolite; furthermore, compared to covalent bonding, physical adsorption helps to maintain the structural conformation of the enzyme [32].

#### 3.2.2. Effect of the Immobilization Parameters on the Enzyme Activity

**Figure 2:**
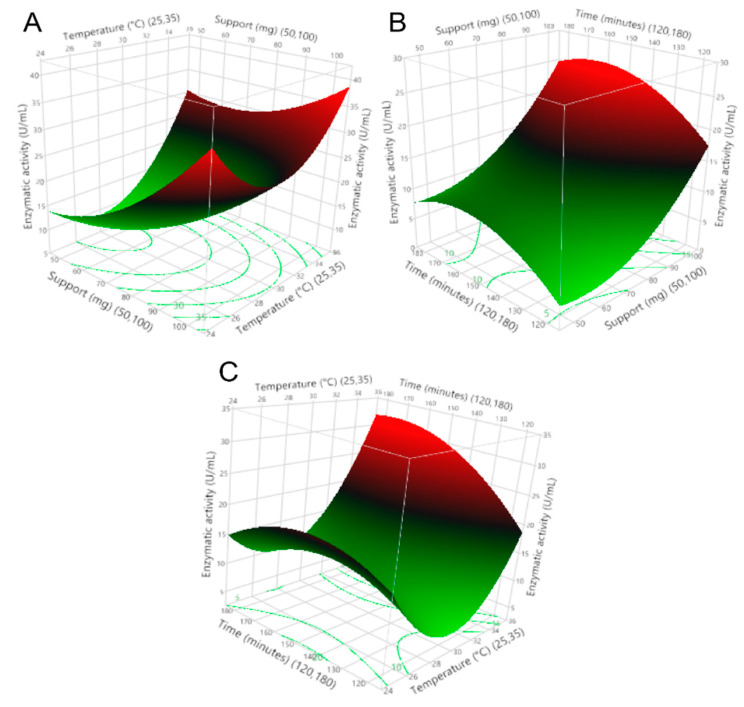
Response surfaces of (**A**) amount support–immobilization temperature vs. enzymatic activity, (**B**) immobilization time–amount support vs. enzymatic activity, and (**C**) immobilization time–immobilization temperature vs. enzymatic activity.

To select the immobilization parameters that provided the maximum enzymatic activity of the enzyme on the zeolite support, as well as to understand the interactions that occurred, immobilization assays were performed, varying the amount of support, temperature, and immobilization time. This research demonstrates a directly proportional relationship between the use of commercial zeolite as a support and the immobilization temperature on the enzymatic activity of the recombinant β-glucosidase. An inversely proportional relationship between enzyme activity and immobilization time was also obtained; this phenomenon is shown in Figure 2.

The regression coefficients of the model are shown in Table 2, indicating the impact of the operating variables and the interaction between the variables on the enzymatic activity. Additionally, the importance of using support was confirmed, as the presence of this zeolite (>50 mg) maintained the enzymatic activity >8 U/mL after the immobilization process. This indicates that the amount of support has a positive effect on the enzymatic activity and the immobilization yield. Furthermore, the enzyme activity remained stable over time and with temperature variations, thus allowing variations in the immobilization process as it seems appropriate.

**Figure 3:**
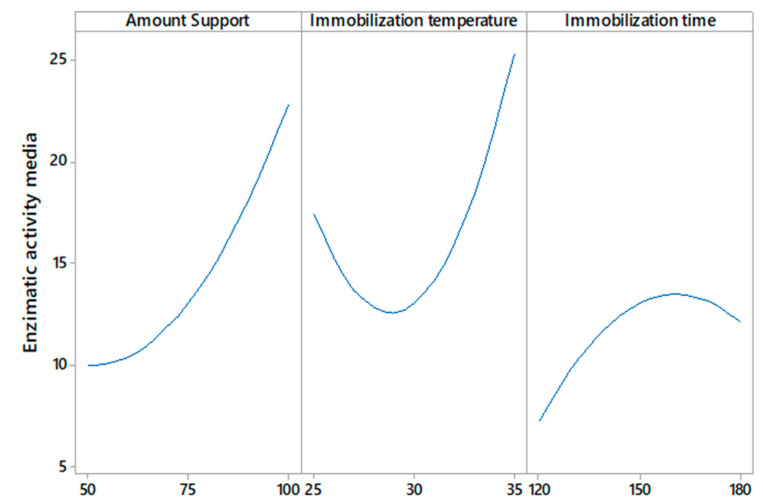
Effect of amount support, immobilization temperature, and immobilization time on enzymatic activity.

On the other hand, Figure 3 shows that any amount of support >50 mg, immobilization temperatures at >30 °C, and a certain range of immobilization time less than 180 min could mitigate the decrease in enzymatic activity above the minimum value. Temperature is one of the most important parameters in the enzyme immobilization process; the results presented in Figure 2 show that the enzyme activity increases by increasing the immobilization temperature from 30 to 35 °C; this indicates that at lower temperatures, the conformation of the enzyme changes. The obtained results agree with a study [33], in which the immobilization of CGTase from *Paenibacillus macerans* on cellulose-coated magnetite microparticles presented the best results when the enzyme was immobilized at high temperatures (40 °C), indicating that at this temperature, the rate of vibrational motion of the enzyme molecules was high. In this way, the statistical software maximization tool was used to obtain the run with the highest enzyme activity, obtaining the result of amount support (100 mg), immobilization temperature (35 °C), and immobilization time (175 min).

### 3.3. Biochemical Characterization

#### 3.3.1. Effect of Temperature and pH on Enzyme Stability

**Figure 4:**
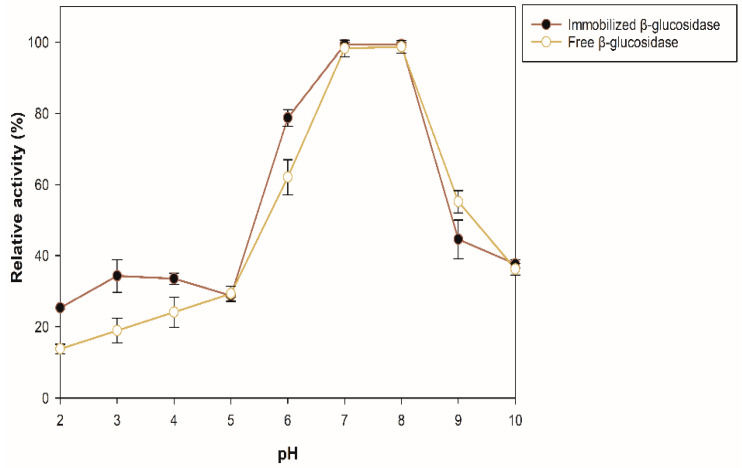
Stability of free and immobilized β-glucosidase at different pH.

The effect of pH and temperature on the enzyme stability was evaluated for the sample with the best enzymatic activity (100 mg of support, 35 °C for 175 min) by quantifying the residual activity after incubating the enzyme preparation for 12 h at 4 °C in buffers of pH 2–10. The percentages of residual activity of the soluble and immobilized β-glucosidase are presented in Figure 4. The immobilized β-glucosidase exhibited greater stability (10–15%) than the free enzyme in the pH range 2–4. At pH 6, the immobilized enzyme is approximately 16% more stable than the free one, and at pH 7 and 8, the same activity was maintained in both, decreasing the activity at alkaline pH.

The molecular mass of proteins is not related to their adsorption efficiency, and it has been demonstrated that the adsorption performance of enzymes to zeolites depends on the pH and increases at or near its isoelectric point (pI) [34]. In this case, the isoelectric point of the recombinant β-glucosidase is 5.63, and the immobilization pH was 5; therefore, its charge is positive, thus allowing to increase the electrostatic interactions and the affinity of adsorption between the enzyme and the zeolite with a negative charge on its surface. Consequently, an increase in the enzymatic activity of the immobilized enzyme compared to the free one can be observed. The immobilization process can modify the stability of the enzyme at different pH, due to the interactions between the enzyme and the support. In addition, media loading can affect the catalytic activity of the immobilized enzyme by causing the pH of its microenvironment to be different from the pH of the reaction medium, either favoring or hindering catalysis [35]. Hence, the immobilization had a positive effect on the enzyme activity, depending on the pH at which activity was measured.

**Figure 5:**
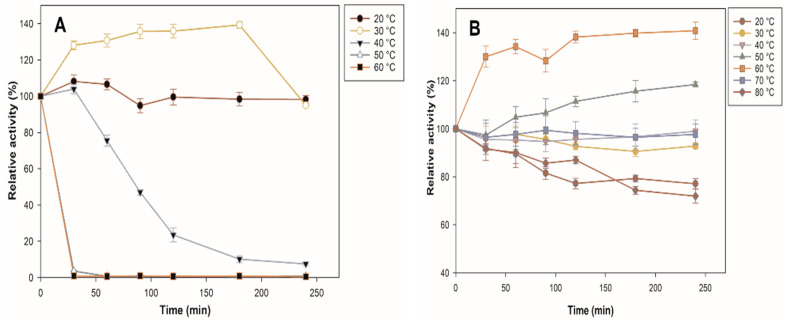
Characterization of β-glucosidase. Effect of temperature on the stability of free (**A**) β and immobilized (**B**) β-glucosidase. All values are the mean of three replicates.

Thermal stability is an important characteristic for the use of the biocatalyst at an industrial level since it could indicate if the processing temperatures are far from their optimum. Figure 5 shows the comparison between the thermal stability profiles of the immobilized and free enzymes. The free enzyme loses 100% of its activity in the first 30 min at 50 and 60 °C, which may be due to irreversible changes in protein conformation, while the immobilized enzyme increases its activity by 18% at 50 °C at 240 min. It stands out that at 60 °C, the immobilized β-glucosidase increases its activity by 40%, and, at 70 °C, it maintains almost 100% of its initial activity; even at 80 °C, it still maintains 72% of its initial activity at 240 min, whereas free enzymes are completely deactivated at the same temperature. The result is consistent with the report by Park et al. [36], who showed that the free enzyme lost >95% of its activity at 65 °C, while immobilization increased enzyme stability. The three-dimensional structure of the immobilized enzyme was stabilized at high temperatures by binding to a solid support [37]. The interaction of the enzyme with the support can rigidify the enzyme structure by inhibiting the conformational freedom and thermal vibration of the polypeptide chain. In the case of an enzyme trapped in porous support (zeolite), the interaction with the pore walls further increases the rigidity of the enzyme structure, thus maintaining its conformation [38]. In addition, it has been shown that selecting silica-based support is a suitable strategy to avoid the thermal denaturation of cellulolytic enzymes [39,40,41].

#### 3.3.2. Enzyme Kinetics

To evaluate the behavior of free and immobilized β-glucosidase against the substrate, a kinetic was performed. Lineweaver–Burk plot was used to determine the K*_M_* and V*_max_* values. The data are the mean value with standard deviation from triplicate experiments. The reaction condition was pH 5.0 (acetate buffer 100 mM) and a temperature of 50 °C. Table 3 shows the kinetic parameters of the immobilized and free β-glucosidase. The immobilized β-glucosidase presented a K*_M_* value of 4.42 mM, which was lower than that of the free enzyme (50.18 mM), indicating that the immobilized enzyme has a higher affinity for *p*-NPG than the free enzyme or that there was an enhanced substrate concentration near the active sites caused by the interactions between the substrate and enzyme-support complex. In some cases, a lower K*_M_* value has been reported after immobilization [42,43]. β-glucosidase is an enzyme that is inhibited by by-product (glucose); it has been reported that immobilization reduces this inhibition by improving K*_M_* values [44]. Different affinity behaviors of the enzyme for the substrate in β-glucosidase immobilized on different supports have been obtained. Ying et al. [45] immobilized β-glucosidase on magnetic Fe_3_O_4_ nanoparticles as a carrier, and these biocatalysts showed a K*_M_* value of 1.77 and 3.12 mmol/L for immobilized and free, respectively. Shan et al. [22] immobilized β-glucosidase thermostable on Na-Y zeolite and found a behavior similar to that reported in this paper with a K*_M_* value of 0.37 and 0.58 mM for immobilized and free, respectively. An increase has also been reported for the immobilization of laccase in zeolite NaY with values of 0.73 and 1.01 for free and immobilized, respectively [21]. The value of K*_M_* found in this research can be attributed to the porous morphology of the zeolite that improves the accessibility of the enzyme by the substrate [46], which contributes to reducing diffusion limitations. In addition, its high surface area maximizes the interactions with the substrate. The V*_max_* value of the immobilized enzyme was lower than the free enzyme; this may occur because the flexibility of the enzyme decreases after immobilization; therefore, there is a decrease in diffusion rates [47]. Similarly, an increase in the turnover number (K_cat_) was observed for the free enzyme, suggesting a greater catalytic activity. However, K_cat_/K*_M_* was determined, which is a parameter to measure catalytic efficiency, obtaining 81.66 for the immobilized β-glucosidase, approximately 10 times higher than that of a free enzyme (8.93). These results indicate a higher catalytic efficiency for the immobilized β-glucosidase, this may be due to a more efficient conformation of the enzyme within the spaces of the support, and the accessibility of the substrate to the active sites of the enzyme might be increased.

#### 3.3.3. Reusability of Immobilized β-glucosidase Test

**Figure 6:**
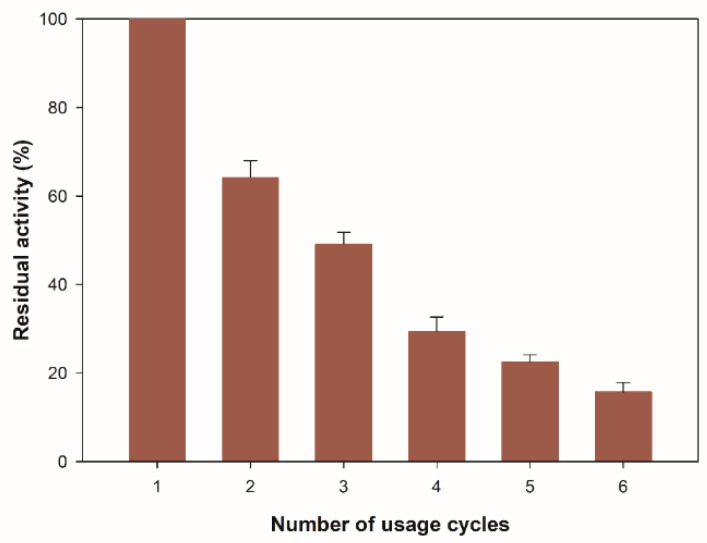
Reusability of immobilized β-glucosidase. All values are the mean of three replicates. Error bars represent the standard deviation within the data set.

To evaluate the possibility of recovering the enzyme from the reaction medium, it is necessary to measure the residual enzyme activity after consecutive reaction cycles because the repeated use of the biocatalyst can balance the costs associated with the production of the enzyme. Figure 6 shows the number of usage cycles for the immobilized β-glucosidase on zeolite. The residual activity after the first cycle was 65% of its initial activity, and after six cycles, it remained at 16%. The decrease in enzyme activity observed after six consecutive cycles of reaction may be due to the gradual leakage and the denaturation of the enzyme, in addition to the mechanism of physical immobilization on the surface of the zeolite structure. Adsorption occurs through the charge in the middle of the first enzyme molecules that diffuse into the porous structure and gradually fill the inner core of the structure by interaction with the silica surface [48,49]. When the pores are full, an excess of the enzyme is established, which does not interact with the surface. These enzyme aggregates are loosely bound to the outer layer of the enzyme adsorbed, and they can be easily leached during the reaction [50], leading to a decrease in catalytic activity during consecutive cycles.

### 3.4. Morphological Analysis

**Figure 7:**
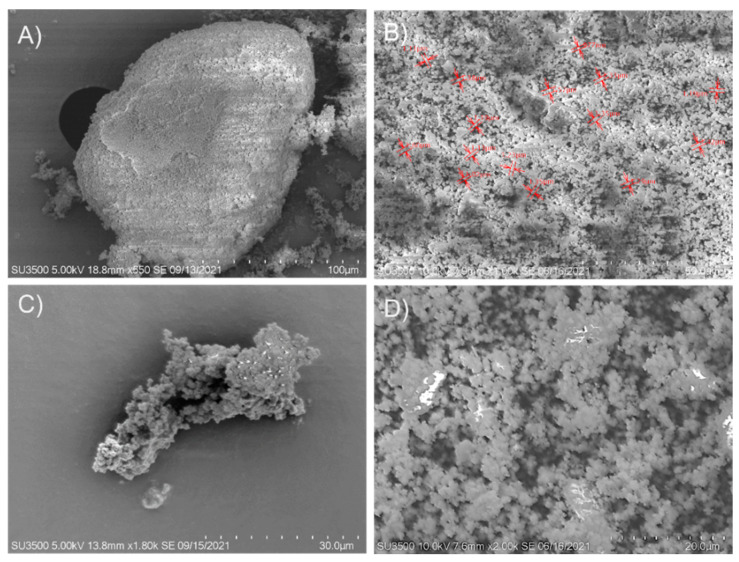
SEM Image of commercial zeolite (**A**,**B**) and recombinant β-glucosidase immobilized on zeolite (**C**,**D**).

The change of morphology of the zeolite that served as a support matrix for β-glucosidase immobilization was determined by scanning electron microscopy. In the zeolite sample (Figure 7A,B), many open pores can be seen; these pores may play a crucial role in enzyme adsorption (presenting advantages such as simplicity, good reproducibility, speed, and no toxic compounds incorporation). The zeolites used have many pores of various sizes ranging from the smallest (less than 1 µm) to larger (20 µm). The micrograph of the immobilized β-glucosidase on zeolite (Figure 7C,D) shows many closed pores; this could be due to the enzyme adsorbed on the surface of the support. Regarding the morphology of the immobilized β-glucosidase, it was packed. The formation of aggregates could be the consequence of covalent bond formations between the reactive groups on the surfaces.

## 4. Conclusions

A protocol for the immobilization of β-glucosidase through adsorption on commercial zeolite was developed and optimized, thus allowing to obtain a novel biocatalyst in solid form with greater thermostability and reusability. The immobilization strategy improved its activity and catalytic efficiency. Parameters that enhance the enzymatic activity of recombinant β-glucosidase after immobilization were obtained. Immobilized β-glucosidase showed a higher catalytic performance through a decrease in the K*_M_*, indicating that a very favorable microenvironment was created inside the pores, improving the catalytic activity of the enzyme. This procedure, based on a simple and fast method, could be applied to other recombinant enzymes rich in lysine and histidine residues on their surface. The results confirmed the potential of the immobilized β-glucosidase for industrial applications with the additional advantages of the use of zeolite.

## Figures and Tables

**Table 1:** Experimental design of the Box–Behnken response surface (amount of support, temperature, and time).

Run	Support (mg)	Temperature (°C)	Time (min)
1	50	25	150
2	100	25	150
3	75	25	120
4	75	25	180
5	50	30	180
6	100	30	180
7	50	30	120
8	100	30	120
9	75	30	150
10	100	35	150
11	50	35	150
12	75	35	120
13	75	35	180

**Table 2:** Response surface regression coefficients: Enzymatic activity vs. amount support, immobilization temperature, and immobilization time (coded coefficients).

Factor	Coef	EE Coef	T Value	*p*-Value	FIV
Intercept	13.06	5.37	2.43	0.093	-
Amount support (mg) (50, 100)	6.41	1.90	3.38	0.043	1.00
Immobilization temperature (°C) (25, 35)	3.92	1.90	2.07	0.131	1.00
Immobilization time (minutes) (120, 180)	2.41	1.90	1.27	0.293	1.00
Amount support (mg) × amount support (mg)	3.35	3.55	0.94	0.414	1.35
Immobilization temperature (°C) × immobilization temperature (°C)	8.33	3.55	2.35	0.100	1.35
Immobilization time (minutes) × immobilization time (minutes)	−3.34	3.55	−0.94	0.417	1.35
Amount support (mg) × immobilization temperature (°C)	−2.46	2.68	−0.92	0.426	1.00
Amount support (mg) × immobilization time (minutes)	0.58	2.68	0.22	0.841	1.00
Immobilization temperature (°C) × immobilization time (minutes)	2.74	2.68	1.02	0.383	1.00

**Table 3:** Kinetic parameters of free and immobilized recombinant β-glucosidase.

Enzyme	K*_M_* (mM)	V*_max_* (µmol/min mg)	K_cat_ (min^−1^)	K_cat_/K*_M_* (min^−1^ mM)
Free β-glucosidase	50.186 ± 3.73	116.28 ± 2.36	448.53 ± 3.47	8.93
Immobilized β-glucosidase	4.42 ± 0.83	60.97 ± 1.91	360.97 ± 2.618	81.66

## Data Availability

Not applicable.

## Sample Availability

Samples of the compounds are available from the authors.
